# Dual AV Nodal Nonreentrant Tachycardia Resulting in Inappropriate ICD Therapy in a Patient with Cardiac Sarcoidosis

**DOI:** 10.1016/s0972-6292(16)30715-x

**Published:** 2014-01-01

**Authors:** Ankur A Karnik, Khashayar Hematpour, Advay G Bhatt, Michael J Mazzini

**Affiliations:** Boston University School of Medicine Cardiology Section, Arrhythmia Service Boston, MA

**Keywords:** supraventricular tachycardia, dual atrioventricular nodal nonreentrant tachycardia, DAVNNT, sarcoidosis

## Abstract

Dual atrioventricular nodal nonreentrant tachycardia (DAVNNT) occurs due to concurrent antegrade conduction over fast and slow atrioventricular nodal pathways and is treated by slow pathway modification. We describe a unique case of a patient with cardiac sarcoidosis who received inappropriate ICD shocks for DAVNNT. Atrial and ventricular device electrograms satisfied both rate and V>A criteria for ventricular tachycardia. We postulate that alterations in refractoriness and conduction as is seen in cardiac sarcoidosis (CS) may have contributed to occurrence of DAVNNT.

## Introduction

Simultaneous antegrade conduction over fast and slow AV nodal pathways was first described by Wu et al. in 1975 [1] and is referred to as a "double fire".[2] This phenomenon may result in DAVNNT. We describe a unique case of a patient with inappropriate ICD therapy for DAVNNT.

## Case report

**Figure 1:**
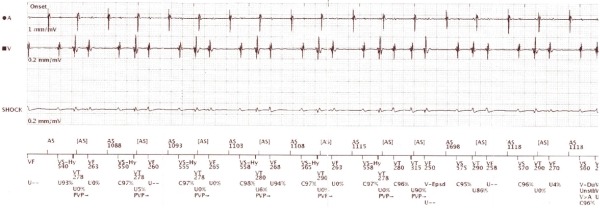
Initial electrograms recorded from ICD telemetry at presentation. Atrial (A) and ventricular (V) near-field electrograms demonstrate an irregular tachycardia with V>A which is interpreted as ventricular tachycardia (VT) by the device in the bottom channel marker.

A 42 year-old man with pulmonary and cardiac sarcoidosis (CS) diagnosed on the basis of mediastinal lymph node biopsy and late gadolinium enhancement on cardiac MRI was referred to an outside institution for dual chamber implantable cardioverter-defibrillator (ICD) for syncope, complete atrioventricular block, and moderate systolic dysfunction (ejection fraction 40%). He was treated with prednisone for three months and atrioventricular conduction subsequently improved. He then presented to a local emergency department after experiencing multiple ICD shocks preceded by palpitations. Device interrogation was interpreted as salvos of non-sustained ventricular tachycardia (Figure 1). He was treated with metoprolol, diltiazem, and amiodarone before transfer to our institution.

**Figure 2:**
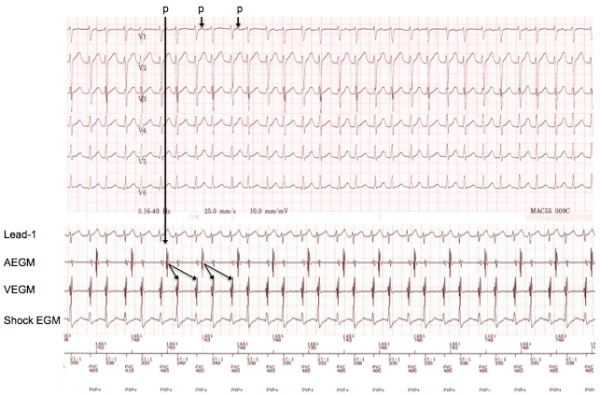
Top panel shows ECG after arrival to the cardiac care unit, with narrow complex tachycardia and visible P wave preceding every other QRS complex. Bottom panel shows concurrent intracardiac tracings through the patient's ICD. Arrows between the atrial and ventricular near-field electrograms indicate a 1:2 response. The channel marker shows an interpretation of ventricular tachycardia by the device.

**Figure 3:**
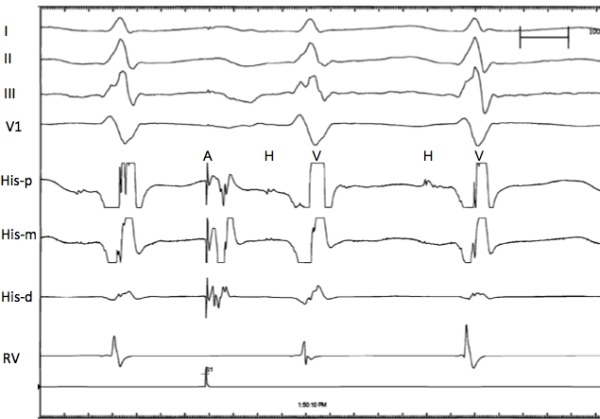
Intracardiac tracings at 200 mm/sec sweep speed recorded during atrial pacing demonstrating an A-HV-HV response. At top are the surface leads followed by His and right ventricular electrogram tracings, and the stimulation channel at the bottom.

Following admission he was noted to have a narrow complex tachycardia with a pattern of grouped beating and P waves visible in a 1:2 pattern; device interrogation with concurrent atrial and ventricular electrograms confirmed 1:2 AV conduction (Figure 2). Ventricular tachycardia and ventricular fibrillation zone cut-offs were 190 and 220 beats per minute respectively treated with anti-tachycardia pacing and biphasic shocks. Ventricular detection enhancements including electrogram morphology match, onset, and stability were programmed on. Device therapies were disabled and he was referred for electrophysiology study, which demonstrated consistent antegrade dual AV nodal conduction during sinus rhythm and with atrial pacing (Figure 3). Ventriculoatrial conduction was absent at a drive train of 600 msec. Atrial effective refractory period (AERP) was 700/210 msec. Slow pathway AV nodal ERP was 700/330 msec. Fast pathway AV nodal ERP was shorter than AERP. He was treated with slow pathway ablation and has experienced no further palpitations or ICD therapies at 12 months follow-up.

## Discussion

Double fire tachycardia is an uncommon and under-recognized entity with approximately 50 cases reported in the literature.[2] Prior reports have identified DAVNNT as a cause of reversible cardiomyopathy and inappropriate referral for pulmonary vein isolation for presumed atrial fibrillation.[2] The authors have previously described a patient with DAVNNT who was misdiagnosed as having inappropriate sinus tachycardia.[3] The differential diagnosis for a narrow complex tachycardia with a P:R ratio of 1:2 includes (1) atrial bigeminy with a low voltage P-wave masked by the preceding T-wave, (2) junctional bigeminy, and (3) atrioventricular nodal re-entrant tachycardia (AVNRT) with 2:1 retrograde block.[4] During electrophysiologic testing, the differential diagnosis includes junctional extrasytole and junction parasystole. The former has a less predictable coupling interval with the preceeding QRS or His potential. The latter is rare and diagnosed when the junctional cycle length is stable and slightly different than the sinus cycle length.[2]

Diagnosis of DAVNNT depends on the ability to demonstrate AV nodal properties. Since the relationship between the His and the fast and slow pathways are stable, a zone of dual ventricular response may be established by altering the atrial cycle length. Quinidine suppresses junctional automaticity and exacerbates DAVNNT while isoproterenol and atropine suppress DAVNNT and exacerbate junctional rhythms.[2] The authors have demonstrated that atrial extrastimuli which terminate tachycardia without His capture effectively rule out junctional bigeminy.[3] Ultimately, definitive proof of DAVNNT is disappearance of the electrophysiologic manifestations of 1:2 AV conduction following slow pathway ablation.[2]

The maintenance of DAVNNT is dependent upon the presence of several unique circumstances. There must be significantly delayed conduction through the slow pathway; the effective refractory period of the conduction system distal to the AV node must be shorter than the difference between the conduction times of the fast and slow pathways; each sinus beat must be delayed sufficiently to allow conduction through the slow pathway to the His-Purkinje system; and retrograde ventriculoatrial conduction should be poor or absent.[2]

The first catheter ablation for DAVNNT was performed in 1994 and is the treatment of choice given the curative potential of ablation and the high failure rate of medical management.[2] Slow pathway ablation may be challenging due to incessant tachycardia as well the similarity of antegrade beats conducted by the slow pathway and junctional ectopy with retrograde block. In these cases, isoproterenol infusion may help by inducing AV conduction exclusively via the fast pathway.[5]

We present the first known case of DAVNNT resulting in inappropriate ICD therapy. In contrast to prior reports, the initial misdiagnosis was based upon interpretation of atrial and ventricular device electrograms which satisfied both rate and V>A criteria for ventricular tachycardia. The timing of ICD implantation for primary prevention of sudden death in CS is controversial, and there is no universally accepted standard of risk stratification in these patients. Electrophysiologic testing has been suggested as a method of risk stratification in asymptomatic patients with imaging evidence of cardiac sarcoid, [6] but there are no data with respect to symptomatic patients or those requiring pacing support. A recent retrospective study also demonstrated a high rate of appropriate and inappropriate shocks in CS patients with ICDs. In that study, patients who received appropriate ICD therapies were more likely to be male, have a history of syncope, lower left ventricular ejection fraction, and need for ventricular pacing.[7] The uncertainty over risk prediction coupled with the high event rate and lack of prospective data is reflected in the current American Heart Association and Heart Rhythm Society Guidelines, which confer a 2A indication for ICD implantation in patients with CS. [8] It is of particular interest that the patient's initial presentation was complete heart block in the setting of cardiac sarcoidosis. We postulate that in our patient, the alterations in refractoriness and conduction necessary for DAVNNT occurred as a consequence of partial AV nodal recovery during prednisone treatment, with residual nodal AV dissociation.
